# Development and validation of a screening model for lung cancer using machine learning: A large-scale, multi-center study of biomarkers in breath

**DOI:** 10.3389/fonc.2022.975563

**Published:** 2022-09-20

**Authors:** Jing Li, Yuwei Zhang, Qing Chen, Zhenhua Pan, Jun Chen, Meixiu Sun, Junfeng Wang, Yingxin Li, Qing Ye

**Affiliations:** ^1^ Laser Medicine Laboratory, Institute of Biomedical Engineering, Chinese Academy of Medical Science and Peking Union Medical College, Tianjin, China; ^2^ Key Laboratory of Weak-Light Nonlinear Photonics, Ministry of Education, School of Physics and TEDA Applied Physics, Nankai University, Tianjin, China; ^3^ Departmentof Cardio-Pulmonary Function, National Clinical Research Center for Cancer, Cancer Institute and Hospital, Tianjin Medical University, Tianjin, China; ^4^ Tianjin Key Laboratory of Lung Cancer Metastasis and Tumor Microenvironment, Tianjin Lung Cancer Institute, Tianjin Medical University General Hospital, Tianjin, China; ^5^ Julius Center for Health Sciences and Primary Care, University Medical Center Utrecht, Utrecht University, Utrecht, Netherlands

**Keywords:** breath analysis, lung cancer, machine learning, PTR-TOF-MS, screening

## Abstract

**Objectives:**

Lung cancer (LC) is the largest single cause of death from cancer worldwide, and the lack of effective screening methods for early detection currently results in unsatisfactory curative treatments. We herein aimed to use breath analysis, a noninvasive and very simple method, to identify and validate biomarkers in breath for the screening of lung cancer.

**Materials and methods:**

We enrolled a total of 2308 participants from two centers for online breath analyses using proton transfer reaction time-of-flight mass spectrometry (PTR-TOF-MS). The derivation cohort included 1007 patients with primary LC and 1036 healthy controls, and the external validation cohort included 158 LC patients and 107 healthy controls. We used eXtreme Gradient Boosting (XGBoost) to create a panel of predictive features and derived a prediction model to identify LC. The optimal number of features was determined by the greatest area under the receiver‐operating characteristic (ROC) curve (AUC).

**Results:**

Six features were defined as a breath-biomarkers panel for the detection of LC. In the training dataset, the model had an AUC of 0.963 (95% CI, 0.941–0.982), and a sensitivity of 87.1% and specificity of 93.5% at a positivity threshold of 0.5. Our model was tested on the independent validation dataset and achieved an AUC of 0.771 (0.718–0.823), and sensitivity of 67.7% and specificity of 73.0%.

**Conclusion:**

Our results suggested that breath analysis may serve as a valid method in screening lung cancer in a borderline population prior to hospital visits. Although our breath-biomarker panel is noninvasive, quick, and simple to use, it will require further calibration and validation in a prospective study within a primary care setting.

## Introduction

Lung cancer (LC) is the largest single cause of death from cancer worldwide (1), and the five-year net survival is in the range of 10–20% for most countries (2). However, early-stage LC is curable, with an overall five-year survival rate of 80% (3). There is therefore an urgency to the development of efficient approaches in the early detection of LC.

Low-dose computed tomography (CT) scanning for the population at high risk is commonly used in LC screening. To reduce cancer mortality, the United States (U.S.) Preventive Services Task Force recommended expanding LC screening to younger individuals and low-intensity smokers (4). However, high false-positive rates, over-diagnosis, limits to applicable coverage, and cumulative radiation exposure remain primary concerns with this type of screening modality (5).

Breath analysis (BA) provides an attractive option (6–8), because the growth of cancer cells is strongly linked to key metabolic pathways that produce detectable amounts of volatile organic compounds (VOCs) in exhaled breath (9). Previous studies have shown that BA can differentiate between LC patients and healthy controls, with an overall accuracy of 69.4% to 100% (10). However, the lack of reproducibility for breath biomarkers among different studies restricts the further implementation of these biomarkers in clinical practice (11). This lack of replicability is primarily because most breath biomarkers were recognized from small pilot studies (the largest study had a sample size of 193), and they lacked independent validation to evaluate their test accuracy (10, 12). Owing to the heterogeneity and variety of physiologic and clinical backgrounds of patients, this deficiency in large-scale samples hinders the development, validation, and implementation of appropriate biomarkers.

Gas chromatography in combination with mass spectrometry and electronic noses are widely used for the investigation of breath biomarkers in LC (10–12). Breath VOCs comprise a very complex matrix that contains a large variety of VOCs at trace amounts (ppbv to pptv) (13, 14). The major flaw of electronic noses in screening the reliable biomarkers of LC is the inferior provision of quantitative results with respect to unknown substances (15, 16). Mass-spectrometric techniques are particularly well suited for biomarker investigations because they offer the possibility of detecting a large variety of compounds of interest with high sensitivity and high accuracy (17–19). However, the commonly encountered issues during conventional gas chromatography in combination with mass spectrometry-based breath-profiling analysis in a large-scale study are the complicated sample-preparation procedure and time-consuming test processes. Direct mass spectrometry—such as selected ion flow tube mass spectrometry and proton transfer reaction-mass spectrometry—are sufficiently sensitive and rapid to allow real-time breath analysis (20, 21). Proton transfer reaction time-of-flight mass spectrometry (PTR-TOF-MS) combines time-of-flight mass spectrometry with a proton transfer flow-drift tube reactor, and provides a high mass-resolving power that enables the separation of isobaric molecules; this allows the measurement of a complete mass spectrum within a fraction of a second (22, 23). Compared with offline sampling such as sample collection into bags or onto traps, online sampling is beneficial in reducing artifacts of sample degradation during collection, storage, and handling or the introduction of impurities. To address the challenges inherent to a large-scale breath study, we herein employed a real-time, sensitive, and reliable analytical instrument, the PTR-TOF-MS, in combination with buffered end-tidal (BET) online sampling (24).

Machine learning-based prediction models have shown promising and even superior predictive performance compared with conventional statistical techniques (25), and the advantages of machine learning in large-scale data processing and its non-linear fitting capability make it particularly useful in resolving medical complications. Therefore, we herein incorporated machine-learning algorithms into the pipeline of LC screening of an individual based on breath-component analysis.

Recent efforts have been undertaken to identify and internally validate LC biomarkers using a relatively small dataset (139 patients with lung cancer and 289 healthy adults), and the results suggested that breath testing may constitute a reliable approach for the detection of LC (26). The goal of the current study, then, was to define and externally validate breath testing for LC screening using breath data from a large number of samples from multiple centers. We therefore exploited a breath test that combined PTR-TOF-MS and a machine-learning algorithm to identify and validate the clinical applicability of our novel biomarkers.

## Materials and methods

This study was conducted and reported in accordance with TRIPOD (27), the guideline for clinical-prediction model studies; and STARD-2015 (28), the reporting guideline for diagnostic test studies. Both checklists were completed and are provided in **e-Tables 1** and **2** in the Supplement.

### Study design and data collection

The dataset comprising biomarker discovery and model development was collected prospectively using a case-control design. Consecutive patients suspected to have LC were prepared for surgery or bronchoscopy in the Pulmonary Oncology Department of the Cancer Institute and Hospital, Tianjin Medical University, and were recruited between February of 2019 and January of 2020. Healthy subjects were enrolled after undergoing health checkups at the Cancer Institute and Hospital, Tianjin Medical University, from April 2019 to May 2019.

The validation dataset was also prospectively collected in a case-control design. Suspected LC patients who were prepared for surgery or bronchoscopy in the Department of Pulmonary Oncological Surgery were recruited from Tianjin Medical University General Hospital between October 2020 and June 2021, and healthy subjects (controls) were recruited from hospital staff of the General Hospital of Tianjin Medical University in November of 2020 and March and December of 2021.

The exclusion criteria for LC patients were those under 18 years of age; patients who showed a history of cancer or a synchronous cancer; or had undergone chemotherapy (with anticancer drugs), immunotherapy, hormonal therapy, or radiotherapy. The exclusion criteria for healthy controls were those under 18 years of age, undergoing pregnancy, individuals with a self-reported history of pulmonary disease, and those manifesting pulmonary nodules confirmed by CT images.

Information regarding a history of lung disease, medication use, fasting, and tobacco smoking was obtained through self-reporting. A history of lung disease was designated as an affirmative response to the question “Have you ever had lung disease?”; use of medications was defined as taking any type of drug (including sprays, pills, capsules, and decoctions) in the previous half-month; an empty stomach was characterized as an affirmative answer to the question “Have you eaten breakfast already?”; and smoking status was delineated as never smoking, being an ex-smoker, of currently smoking. Smoking denoted at least one cigarette every day, which continued to or averaged over six or more months; and an ex-smoker quit smoking four or more months prior to sampling. We determined the amount of smoking by counting the number of cigarettes smoked per day.

### Calculation of sample size

In concert with the recommendations of TRIPOD and PROBAST regarding sample-size calculation, we determined the sample size needed for developing and validating the respective models. The sample size for model development was ascertained with the method recently proposed by Riley et al. (29), as well as using 10 events per variable (EPV) as a rule-of-thumb. We set Cox-Snell’s adjusted R (2) to 0.1 and the desired shrinkage equal to 0.9 as recommended. Since machine-learning models may require additional data relative to fitting a statistical model, we added a conservative factor of 10%. Based on our calculations, the desired sample size for model development was 1868, with a conservative adjustment to 2055 (i.e., 1868*1.1). Cases and controls were collected at a ratio of approximately 1:1, and with 22 candidate variables our EPV was 47 (i.e., 2055*0.5/22), which was far larger than that required using rule-of-thumb.

We computed the sample size for model validation according to a requirement of at least 100 patients in both groups, with and without the outcome of interest (i.e., primary LC).

### Outcome and reference standards

We obtained samples of lung-tissue lesions from LC patients by bronchoscopy or surgery for pathologic examination, and clinical status (including stage and type of LC) was confirmed by pathologic diagnosis within one month after sampling. The disease status of healthy controls was determined by physical examination; i.e., individuals younger than 45 years of age underwent lung X-rays while individuals older than 45 underwent either lung X-rays or lung CT scans.

### PTR–TOF-MS analysis

PTR-TOF-MS (PTR-TOF-MS 1000, Ionicon Analytik GmbH, Innsbruck, Austria) offers quantitative analysis of the entire mass range (1–10,000 amu) within split-seconds and with an ultra-low detection limit (LoD<10 pptv) and high resolution (>2000 m/△m). The BET-sampling system (Ionicon Analytik GmbH, Innsbruck, Austria) also affords the two distinct advantages of collecting the end-tidal fraction of exhaled breath gas and maintaining a normal breathing pattern for test subject after one exhalation. This system allows the measurement of endogenous compounds originating from the alveolar blood-gas exchange, and reduces the risk of hyperventilation.

Our procedure was as follows. The test subject exhaled directly into the buffer tube of the BET-sampling system equipped with a disposable and sterile mouthpiece (Polypropylene; Art. Nr. 31-30-0022, Germany) and the procedure was repeated three times. The buffer tube was maintained at 80°C by a heating system so as to eliminate the effect of condensation of humidified breath gas, and the collected gas was introduced into the ionization section by the inlet line of the instrument. The ionized molecules were then separated by their mass-to-charge ratio (m/z) and subsequently detected. The pressure and temperature in the drift tube were 2.3 mbar and 70°C, respectively, with an electric drift field of 600 V. A total of 318 features (m/z) were thus extracted from the acquired spectrum of each exhaled breath sample.

### Data analysis

#### Candidate predictors

Raw PTR-MS spectra were acquired using the data acquisition software loniTOF30. Data were preprocessed to extract all features that were organic compounds and expiratory concentrations that were higher than the respective inspiratory concentrations. Twenty-two features of endogenous VOCs that were ultimately determined for all test subjects in the discovery dataset were chosen as candidate features.

#### Feature selection

Before feature selection we first standardized our dataset with an estimated mean and variance from the training set (standardization of external validation set was also based on mean and variance from the training set), and to further reduce the candidate-feature set, we calculated the Spearman correlation between each pair of features. We then randomly removed one of the features from a pair with a correlation greater than 0.99, and this resulted in a final candidate set of 14 features. Finally, we ran an eXtreme Gradient Boosting (XGBoost) classifier (30) and ranked the remaining 14 features using the inherent feature importance from the classifier.

#### Model selection

The XGBoost models were iteratively trained with the feature subset ranked at the top, starting with the most important feature and with one feature added each time. At completion, we respectively compared 14 models with 1 to 14 features; and model performance was evaluated *via* a 10-fold cross validation. The area under the receiver‐operating characteristic (ROC) curve (AUC) averaged over 10 validation results was used as our criterion for model selection. To achieve a balance between model performance and simplicity, we set the performance-reduction tolerance at 1%, indicating that the minimal model performance requirement was 99% of the highest AUC among the 14 models. We then chose the final model that met both the minimal performance requirement and that possessed the fewest features.

#### Statistical analysis of model performance

Continuous variables are expressed as median and inter-quartile ranges (IQRs), and categorical variables are expressed as counts and percentages. The discrimination of the predictive model was assessed using ROC curves and AUCs, while calibration was assessed with the calibration curve.

We also calculated diagnostic performance measures—including sensitivity, specificity, precision, recall, and accuracy—based upon confusion matrix with a pre-specified positivity threshold of 0.5.

The implementations of our feature engineering process, predictive model development, and validation were based on Python Scikit-learn 0.22.1 (31).

### Ethics approval

The study was conducted according to the guidelines of the Declaration of Helsinki and approved by the Ethics Committees of the Cancer Institute and Hospital, Tianjin Medical University; and Tianjin Medical University General Hospital. The present trial was registered with the Institutional Review Board of the Chinese Clinical Trial Registry (registration number: chiCTR1900023659), and all methods were conducted in accordance with relevant guidelines and regulations. Informed consent was obtained from all participants.

## Results

### Description of the derivation and validation datasets

**Figure 1:**
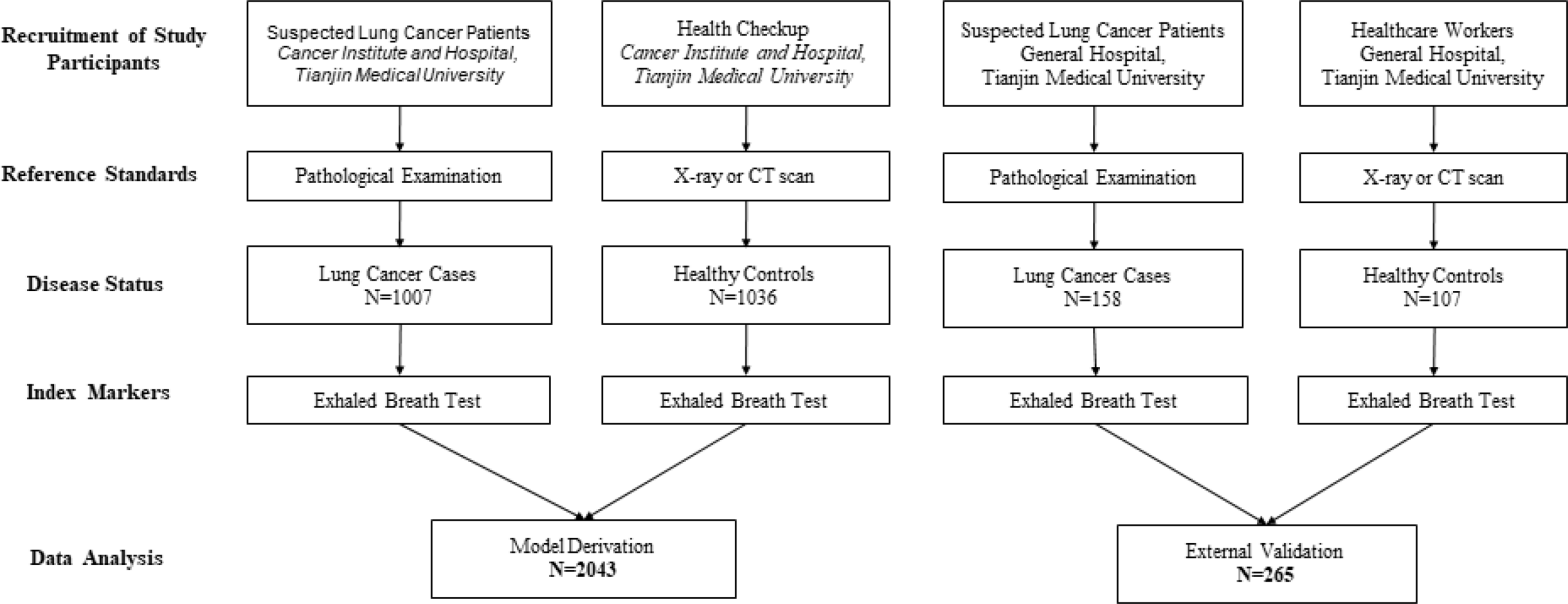
Flow chart for patient recruitment in model development and validation cohorts.

The flow chart for patient recruitment is shown in **Figure 1**. For model derivation, we recruited a total of 2043 participants, including 1007 patients with primary LC and 1036 healthy controls from the Cancer Institute and Hospital, Tianjin Medical University. Mean age of the 1007 patients with primary LC (559 males, 55.51%) was 61 years (age range, 21–81 years), and the most-common smoking status of the patients was non-smoker—accounting for 45.08%. The principal tumor cell types were adenocarcinoma (62.36%), squamous cell carcinoma (15.89%), and small-cell carcinoma (9.04%). At the time of LC diagnosis, we noted 273 patients with stage I disease (27.11%), 121 with stage II (12.02%), 128 patients with stage IIIIV (12.71%), and 170 patients with stage IV (16.88%). Of the enrolled patients, 387 (38.43%) reported that they were fasting at the time of breath sampling. The mean age of the 1036 healthy controls (536 males, 51.74%) was 45 years (age range, 22–90 years), 776 of the subjects were non-smokers (74.91%), and 857 (82.72%) were fasting at the time of breath sampling.

The independent-validation cohort comprised 265 subjects (including 158 patients with primary LC and 107 healthy controls) who came from the General Hospital of Tianjin Medical University. Mean age of the 158 patients with primary LC (63 males, 39.87%) was 63 years (age range, 33–78 years), and the most-common smoking status was smoker—accounting for 49.37%. At the time of LC diagnosis, we noted 133 patients with adenocarcinoma (84.18%), 15 with squamous cell carcinoma (9.49%), and four with small-cell lung cancer (2.53%). Of the enrolled patients, 17 (10.76%) reported that they were fasting at the time of breath sampling. The mean age of the 107 healthy controls (40 males, 37.38%) was 30 years (age range, 19–74 years), 94 of the subjects were non-smokers (87.85%), and nine (8.41%) were fasting at the time of breath sampling (the baseline characteristics of these individuals are shown in **Table 1**).

**Table 1:** Baseline characteristics of the individuals included in the study.

	Derivation		Validation	
	Lung cancer (n = 1007)	Healthy control (n = 1036)	Lung cancer (n = 158)	Healthy control (n = 107)
Male (%)	559 (55.51%)	536 (51.74%)	63 (39.87%)	40 (37.38%)
Age (IQR) [Range]	61 (54, 66) [21-81]	45 (35, 58) [22-90]	63 (58, 69) [33-78]	30 (24, 43)[19-74]
Smoking (%)				
Smokers	382 (37.94%)	209 (20.17%)	78 (49.37%)	12 (11.22%)
Ex-smokers	171 (16.98%)	51 (4.92%)	24 (15.19%)	1 (0.93%)
Non-smokers	454 (45.08%)	776 (74.91%)	56 (35.44%)	94 (87.85%)
BMI(IQR)	24.03 (22.04, 26.30)	24.06 (21.97, 26.30)	23.96 (21.64, 25.92)	22.48 (20.28, 25.45)
Fasting (%)	387 (38.43%)	857 (82.72%)	17 (10.76%)	9 (8.41%)
Adenocarcinoma (%)	628 (62.36%)	NA	133 (84.18%)	NA
Squamous cell carcinoma (%)	160 (15.89%)	NA	15 (9.49%)	NA
Small-cell lung cancer (%)	91 (9.04%)	NA	4 (2.53%)	NA
Missing	128 (12.71%)		6 (3.80%)	
Stage (%)				
0	31 (3.08%)	NA	–	NA
I	273 (27.11%)	NA	–	NA
II	121 (12.02%)	NA	–	NA
III	128 (12.71%)	NA	–	NA
IV	170 (16.88%)	NA	–	NA
Missing	284 (28.20%)			

NA, Not applicable for healthy control group.

### Development and validation of the prediction model

#### Feature selection and importance ranking

**Figure 2:**
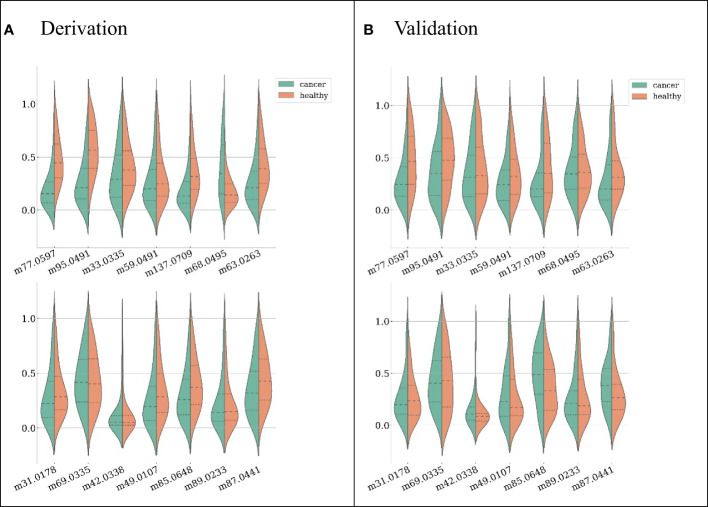
Feature distributions on the derivation dataset **(A)** and validation dataset **(B)**, ranked by their importance (the first feature from left on the first row is the most important). For each feature, both distributions from LC patients (green) and healthy subjects (red) are shown.

Candidate features were first selected by their pairwise correlations, and 14 features were retained for subsequent data analysis. The features selected and their distributions are depicted in **Figure 2** (ranked by their importance), where green represents LC patients and red represents healthy subjects.

**Figure 3:**
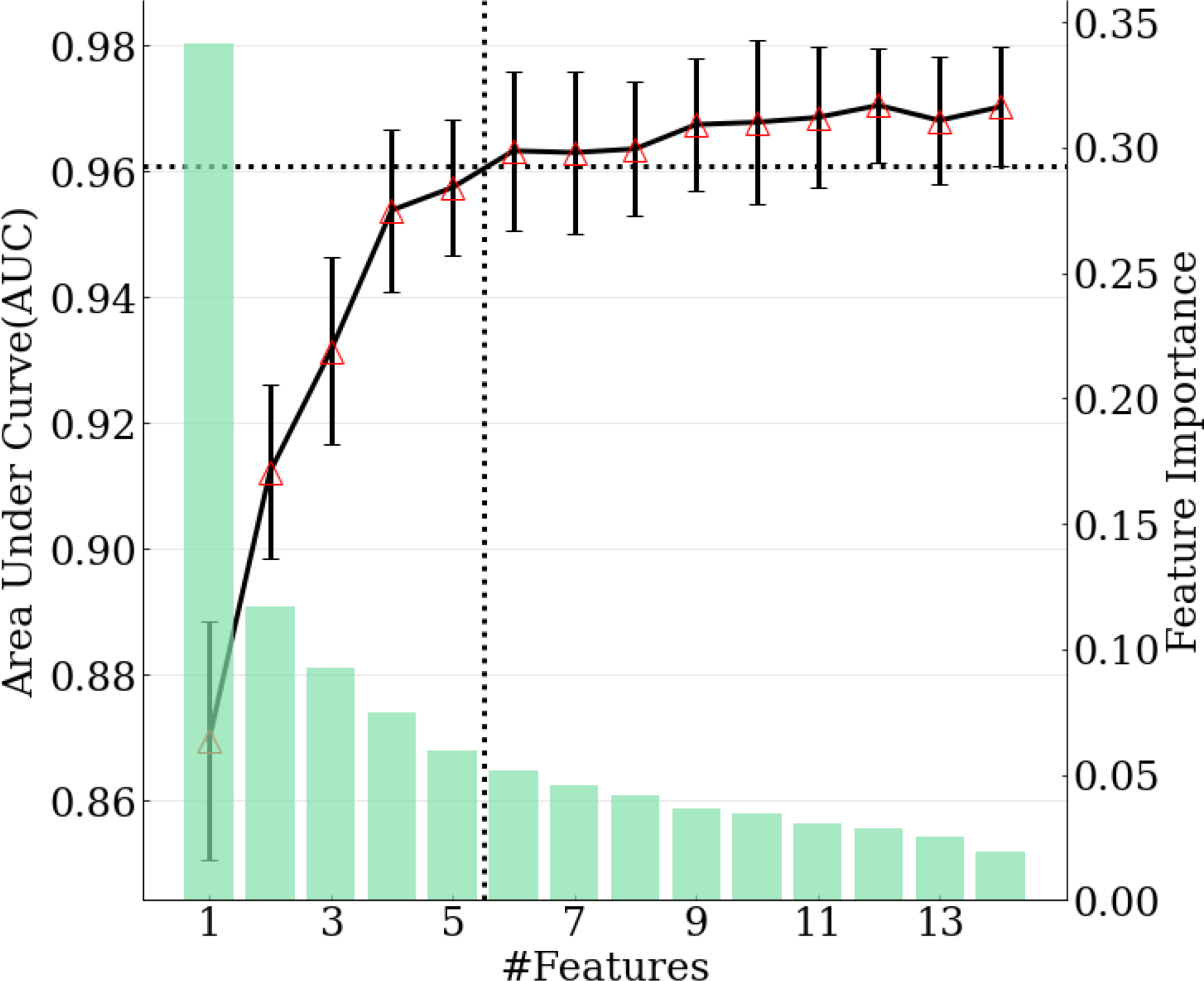
Relation between number of features selected in the model and model performance. Green bars correspond to feature importance. Black solid line corresponds to AUC calculated with top 1-14 features. Black dotted lines demonstrate the number of features selected when achieving 99% of maximum AUC.

The model achieving the greatest AUC included the top 12 features, and it yielded an AUC (averaged across 10-fold cross-validation) of 0.970. Thus, the minimal performance requirement was 0.961 (i.e., 99%*0.970). The model with the fewest features that met this requirement was selected as the final model, and it included the top six features, with an AUC of 0.963. (**Figure 3**) The features included in the ultimate model were ‘m77.0597 ([C_3_H_8_O_2_] H+)’, ‘m95.0491 ([C_6_H_6_O] H+)’, ‘m33.0335 ([CH_4_O] H+)’, ‘m59.0491 ([C_3_H_6_O] H+)’, ‘m137.0709 ([C_7_H_8_N_2_O] H+)’, and ‘m68.0495 ([C_4_H_5_N] H+)’.

**Figure 4:**
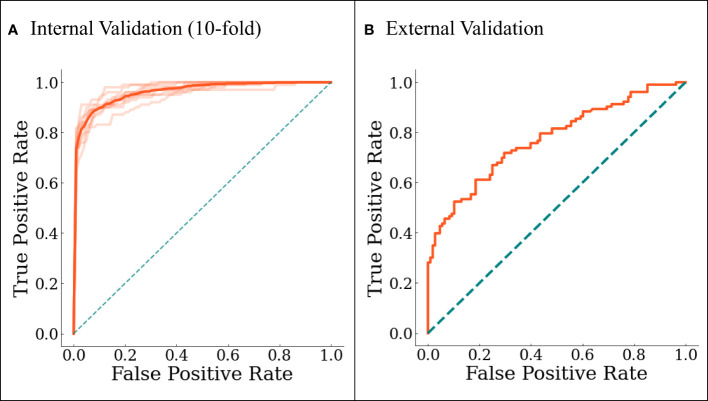
The ROC curves for **(A)** internal 10-fold cross-validation and **(B)** external validation. In **(A)**, darker line represents the averaged results.

**Figure 5:**
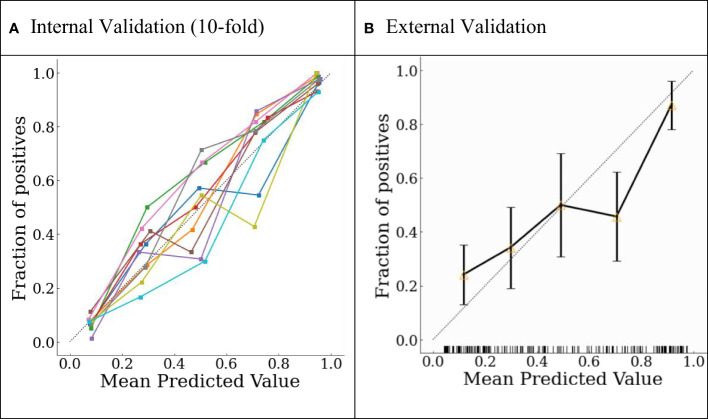
Probability calibration plots for **(A)** internal 10-fold cross-validation and **(B)** external validation.

The final model was internally validated with 10-fold validation and externally validated with the independent-validation dataset, and the AUCs were 0.963 and 0.771 for internal and external validations, respectively (**Figure 4**). The calibration curves showed acceptable alignment for both the derivation and validation datasets (**Figure 5**).

With a predefined positivity threshold of 0.5, the sensitivity and specificity of the final model were 87.1% and 93.5% for the derivation data, respectively; and 67.7% and 73.0% for the validation data, respectively. The confusion matrix (**Table 2**) and diagnostic-accuracy measures for both the derivation and validation datasets are provided in **Table 3**.

**Table 2:** Confusion matrix of the derivation and validation datasets.

Derivation data		Lung cancer diagnosed by the current gold standard		Total
		Present	Absent	
Model prediction	Positive	True positive = 877	False positive = 67	944
	Negative	False negative= 130	True negative = 969	1099
Total		1007	1036	2043
**Validation data**		Lung cancer diagnosed by the current gold standard		Total
		Present	Absent	
Model prediction	Positive	True positive = 107	False positive = 29	136
	Negative	False negative= 51	True negative = 78	129
Total		158	107	265

**Table 3:** Model performance of diagnostic accuracy in the derivation and validation datasets.

	Training (95% CI)	Validation (95% CI)
AUC	0.963 (0.941–0.982)	0.771 (0.718–0.823)
Accuracy	0.904 (0.888–0.925)	0.704 (0.654–0.753)
Sensitivity/Recall	0.871 (0.822–0.926)	0.677 (0.598–0.750)
Specificity	0.935 (0.884–0.967)	0.730 (0.660–0.798)
PPV/Precision	0.930 (0.883–0.961)	0.706 (0.631–0.779)
F-score	0.899 (0.880–0.924)	0.690 (0.625–0.750)

#### Sensitivity and subgroup analyses on internal and external validation

In the sensitivity analyses, we evaluated the performance of the developed model in distinguish different histological subtypes from healthy controls, and also evaluated the model performance in cancer staging. In the subgroup analyses, we evaluated the model performance in gender groups (male and female), age groups (<45 and 45 years old), fasting groups, and smoking groups (evaluating the model performance in ever-smoking and non-smoking groups separately). The model performance is consistent with the overall results in the sensitivity and subgroup analysis. All these results are provided in **e-Tables 3**, **4** and **5** in the Supplementary.

## Discussion

In the nearly 50 years since Linus Pauling first demonstrated the presence of VOCs in human breath, investigators have published over 50 reports showing a strong biologic rationale for using breath biomarkers in the detection of LC. Nevertheless, prior to reaching the clinical setting, this promising approach still faces the challenges of the inconsistent biomarkers exhibited in previous studies: these include limited study cohorts, single study sites, and a lack of validation.

In the current large-scale, multi-center biomarker study, efforts were made to define more reliable breath biomarkers. We first recruited a large cohort of 2043 subjects and analyzed their breaths to develop a predictive panel using machine learning so as to reduce the influence of patient-related individual differences. We thus designed a real-time, sensitive, and reliable instrument coupled with BET sampling for the online collection of alveolar air to reduce the influence of sampling and environmental confounders. Through the exploitation of the machine-learning algorithm XGboost, a panel of six features was defined that revealed an AUC of 0.963, a sensitivity of 87.1%, and a specificity of 93.5%. Second, our panel was validated from a dataset measured at a different hospital, and which achieved an AUC of 0.771. These data of large-scale breath testing and machine learning exhibited the potential to overcome the methodologic challenges of breath tests in the detection of LC, and showed that our metabolic breath panel generated a strong potential for application as a screening tool in clinical practice for the detection of LC.

The PTR-TOF-MS is one of the most powerful techniques for online monitoring of trace VOCs, it can detect mass spectrum peaks with m/z less than 500 and simultaneously achieved accurate concentration of these features, while the peak intensity as a substitute indicator for concentration was used in most mass spectrum techniques. In addition, it has a low detection limit of 10 ppt and a wide detection linear range of 5 orders of magnitude. These characteristics make PTR-TOF-MS hold potentially great value for model development of cancer identification. In this study, we developed the model based on the features extracted from PTR-TOFMS data. The identified VOCs based on m/z was 1,3-Propanediol, phenol, methanol, acetone, m-aminobenzamide and butene nitrile according to the library established based on PTR-TOF-MS. Alcohols and ketones are most commonly detected compounds as biomarkers as lung cancer (32). The formation of some alcohols has been repeatedly reported in the literature to be associated with the growth and metastasis of cancer, suggesting the existence of significance of alcohols in indicating lung cancer (33). Acetone can be produced from the spontaneous decarboxylation of acetoacetate, and it has been used as a biomarker for activation of ketone metabolism, which suggesting that metabolism of ketone bodies might be important for lung cancer cells. It has been confirmed that when there is cancer cell activity in the body, abnormal cell proliferation triggers a stress response that causes increased secretion of adrenocorticotropic hormones (monohydroxy phenolics) in the body (34), suggesting that phenolic metabolites may have an indicator role for lung cancer. In addition, m-aminobenzamide and butene nitrile have not been reported in the literature.

There were, however, some limitations to our study. First, we employed a population-based, case-control design for recruiting participants, individuals with pulmonary nodules confirmed by CT images were excluded from healthy controls, which may lead to overestimate of the predictor–outcome association as well as the model performance. Although a phase-3 analysis (such as model development) was executed, our study can only be viewed as a phase-2 study for biomarker exploration according to the definition provided by Pepe et al. (35). We thus plan a follow-up phase-3 study with nested case-controls within a population cohort to confirm the performance of the proposed markers, and to validate the model in a real-world setting. Second, only 304 cases (30.19% of all LC cases) were diagnosed with early-stage LC in the derivation dataset (stage 0 + stage I), which can differ from the screening setting. Third, the two centers involved in this study were from the same city, which may have limited the robustness of the panel in general clinical practice. Finally, whether the breath panel established in this study excluded the interference of other lung diseases in otherwise healthy individuals requires further verification in the future. Alternatively, a new expiratory database that includes other lung diseases could be implemented. Since the breath panel was selected based upon machine learning, we also propose that our analysis will emerge as more robust when additional participating centers and individuals are recruited to the study.

In summary, we identified a breath-biomarker panel consisting of six features that was defined and validated as an effective tool for the detection of LC in a multi-center phase-2 study. The biomarker panel was applied to discriminate patients with LC from a healthy population (without LC), and whose screening performance was externally validated. This breath panel showed a robust potential for LC screening in clinical practice. However, additional prospective data are needed within a cohort-study design in a primary care setting where the prevalence of LC would be far lower, so as to confirm the validity of our findings and to establish the optimal predictive model.

## Data availability statement

These data are not publicly available due to concerns for subject privacy. Requests to access the data can be directed to the corresponding author.

## Ethics statement

The study was conducted according to the guidelines of the Declaration of Helsinki and approved by the Ethics Committees of the Cancer Institute and Hospital, Tianjin Medical University and Tianjin Medical University General Hospital. The present trial was registered with the Institutional Review Board of the Chinese Clinical Trial Registry (registration number: chiCTR1900023659), and all methods were conducted in accordance with relevant guidelines and regulations. Informed consent was obtained from all participants. The patients/participants provided their written informed consent to participate in this study. Written informed consent was obtained from the individual(s) for the publication of any potentially identifiable images or data included in this article.

## Author contributions

MS took responsibility for (i.e., is the guarantor of) the content of the manuscript, including the data and analysis. JL performed the experiments and assisted with manuscript writing. YZ and QY performed computational data analyses. QC provided samples for model derivation. ZP and JC provided samples for the validation cohort. MS conceived and designed the study, established collaborations, wrote the manuscript, and allocated funding for this study. JW provided critical technical assistance and consultation, and reviewed the manuscript. YL established collaborations and provided scientific direction. All authors contributed to the article and approved the submitted version.

## Funding

This work was supported by the Chinese Academy of Medical Sciences Initiative for Innovative Medicine (2018-I2M-AI-012).

## Acknowledgments

We wish to thank all of the participants included in this study.

## Conflict of interest

The authors declare that the research was conducted in the absence of any commercial or financial relationships that could be construed as a potential conflict of interest.

## Publisher’s note

All claims expressed in this article are solely those of the authors and do not necessarily represent those of their affiliated organizations, or those of the publisher, the editors and the reviewers. Any product that may be evaluated in this article, or claim that may be made by its manufacturer, is not guaranteed or endorsed by the publisher.

## Abbreviations

AUC, area under the receiver‐operating characteristic curve; BA, Breath analysis; BET, buffered end-tidal; EPV, events per variable; IQR, inter-quartile range; LC, lung cancer; PTR-TOF MS, proton transfer reaction-time of flight-mass spectrometry; XGBoost, eXtreme Gradient Boosting; VOCs, volatile organic compounds.
