# Supplementary material for: Development and validation of a screening model for lung cancer using machine learning: A large-scale, multi-center study of biomarkers in breath
Source: Front Oncol. 2022 Sep 20;12:975563. doi: 10.3389/fonc.2022.975563 (PMC9531270; doi:10.3389/fonc.2022.975563)
Supplement: Supplementary file 1 [file DataSheet_1.docx]

**Supplementary Information**

**Development and Validation of a Screening Model for Lung Cancer with Machine Learning: A Large-Scale, Multi-center Phase 2 Study of Breath Biomarkers**

**Jing Li, Yuwei Zhang, Qing Chen, Zhenhua Pan, Jun Chen，Meixiu Sun, Junfeng Wang, YingxinLi, Qing Ye**

**Supplementary e-Table 1. Checklist of items to include when reporting a study developing or validating a multivariable prediction model for diagnosis or prognosis**[**^a^**](https://www.ncbi.nlm.nih.gov/pmc/articles/PMC4454817/table/tbl1/?report=objectonly#t1-fn1)

| **Section/topic** | **Item** | **Development or validation?** | **Checklist item** | **Page** |
| --- | --- | --- | --- | --- |
| **Title and abstract** | | | | |
| Title | 1 | D;V | Identify the study as developing and/or validating a multivariable prediction model, the target population, and the outcome to be predicted. | 1 |
| Abstract | 2 | D;V | Provide a summary of objectives, study design, setting, participants, sample size, predictors, outcome, statistical analysis, results, and conclusions. | 2-3 |
| **Introduction** | | | | |
| Background and objectives | 3a | D;V | Explain the medical context (including whether diagnostic or prognostic) and rationale for developing or validating the multivariable prediction model, including references to existing models. | 4-6 |
|  | 3b | D;V | Specify the objectives, including whether the study describes the development or validation of the model, or both. | 6-7 |
| **Methods** | | | | |
| Source of data | 4a | D;V | Describe the study design or source of data (e.g., randomised trial, cohort, or registry data), separately for the development and validation data sets, if applicable. | 7-8 |
|  | 4b | D;V | Specify the key study dates, including start of accrual; end of accrual; and, if applicable, end of follow-up. | 7-8 |
| Participants | 5a | D;V | Specify key elements of the study setting (e.g., primary care, secondary care, general population) including number and location of centres. | 7-8 |
|  | 5b | D;V | Describe eligibility criteria for participants. | 8 |
|  | 5c | D;V | Give details of treatments received, if relevant. | 8 |
| Outcome | 6a | D;V | Clearly define the outcome that is predicted by the prediction model, including how and when assessed. | 9-10 |
|  | 6b | D;V | Report any actions to blind assessment of the outcome to be predicted. | N/A |
| Predictors | 7a | D;V | Clearly define all predictors used in developing the multivariable prediction model, including how and when they were measured. | 10-11 |
|  | 7b | D;V | Report any actions to blind assessment of predictors for the outcome and other predictors. | N/A |
| Sample size | 8 | D;V | Explain how the study size was arrived at. | 9 |
| Missing data | 9 | D;V | Describe how missing data were handled (e.g., complete-case analysis, single imputation, multiple imputation) with details of any imputation method. | N/A |
| Statistical analysis methods | 10a | D | Describe how predictors were handled in the analyses. | 11-12 |
|  | 10b | D | Specify type of model, all model-building procedures (including any predictor selection), and method for internal validation. | 12 |
|  | 10c | V | For validation, describe how the predictions were calculated. | 12 |
|  | 10d | D;V | Specify all measures used to assess model performance and, if relevant, to compare multiple models. | N/A |
|  | 10e | V | Describe any model updating (e.g., recalibration) arising from the validation, if done. | N/A |
| Risk groups | 11 | D;V | Provide details on how risk groups were created, if done. | N/A |
| Development *vs* validation | 12 | V | For validation, identify any differences from the development data in setting, eligibility criteria, outcome, and predictors. | 7-12 |
| **Results** | | | | |
| Participants | 13a | D;V | Describe the flow of participants through the study, including the number of participants with and without the outcome and, if applicable, a summary of the follow-up time. A diagram may be helpful. | 13-14，26-28 |
|  | 13b | D;V | Describe the characteristics of the participants (basic demographics, clinical features, available predictors), including the number of participants with missing data for predictors and outcome. | 13-14 |
|  | 13c | V | For validation, show a comparison with the development data of the distribution of important variables (demographics, predictors, and outcome). | 13-14 |
| Model development | 14a | D | Specify the number of participants and outcome events in each analysis. | 14-16 |
|  | 14b | D | If done, report the unadjusted association between each candidate predictor and outcome. | N/A |
| Model specification | 15a | D | Present the full prediction model to allow predictions for individuals (i.e., all regression coefficients, and model intercept or baseline survival at a given time point). | 15-16 |
|  | 15b | D | Explain how to use the prediction model. | N/A |
| Model performance | 16 | D;V | Report performance measures (with CIs) for the prediction model. | 15-16 |
| Model updating | 17 | V | If done, report the results from any model updating (i.e., model specification, model performance). | N/A |
| **Discussion** | | | | |
| Limitations | 18 | D;V | Discuss any limitations of the study (such as nonrepresentative sample, few events per predictor, missing data). | 17-18 |
| Interpretation | 19a | V | For validation, discuss the results with reference to performance in the development data, and any other validation data. | 17 |
|  | 19b | D;V | Give an overall interpretation of the results, considering objectives, limitations, results from similar studies, and other relevant evidence. | 17-18 |
| Implications | 20 | D;V | Discuss the potential clinical use of the model and implications for future research | 18 |
| **Other information** | | | | |
| Supplementary information | 21 | D;V | Provide information about the availability of supplementary resources, such as study protocol, Web calculator, and data sets. | 7 |
| Funding | 22 | D;V | Give the source of funding and the role of the funders for the present study. | 19 |

^a^Items relevant only to the development of a prediction model are denoted by *D*, items relating solely to a validation of a prediction model are denoted by *V*, and items relating to both are denoted *D;V*. We recommend using the TRIPOD Checklist in conjunction with the TRIPOD explanation and elaboration document.

**Supplementary e-Table 2. Standards for Reporting Diagnostic accuracy studies**

| **Section & Topic** | **No** | **Item** | **Reported on page #** |
| --- | --- | --- | --- |
| **TITLE OR ABSTRACT** |  |  |  |
|  | **1** | Identification as a study of diagnostic accuracy using at least one measure of accuracy  (such as sensitivity, specificity, predictive values, or AUC) | 2 |
| **ABSTRACT** |  |  |  |
|  | **2** | Structured summary of study design, methods, results, and conclusions (for specific guidance, see STARD for Abstracts) | 2-3 |
| **INTRODUCTION** |  |  |  |
|  | **3** | Scientific and clinical background, including the intended use and clinical role of the index test | 4-6 |
|  | **4** | Study objectives and hypotheses | 6-7 |
| **METHODS** |  |  |  |
| *Study design* | **5** | Whether data collection was planned before the index test and reference standard were performed (prospective study) or after (retrospective study) | 7-8 |
| *Participants* | **6** | Eligibility criteria | 8 |
|  | **7** | On what basis potentially eligible participants were identified (such as symptoms, results from previous tests, inclusion in registry) | 8-9 |
|  | **8** | Where and when potentially eligible participants were identified (setting, location and dates) | 7-8 |
|  | **9** | Whether participants formed a consecutive, random or convenience series | 7-8 |
| *Test methods* | **10a** | Index test, in sufficient detail to allow replication | 7-13 |
|  | **10b** | Reference standard, in sufficient detail to allow replication | 9-10 |
|  | **11** | Rationale for choosing the reference standard (if alternatives exist) | 9-10 |
|  | **12a** | Definition of and rationale for test positivity cut-offs or result categories of the index test, distinguishing pre-specified from exploratory | N/A |
|  | **12b** | Definition of and rationale for test positivity cut-offs or result categories of the reference standard, distinguishing pre-specified from exploratory | N/A |
|  | **13a** | Whether clinical information and reference standard results were available to the performers/readers of the index test | N/A |
|  | **13b** | Whether clinical information and index test results were available to the assessors of the reference standard | N/A |
| *Analysis* | **14** | Methods for estimating or comparing measures of diagnostic accuracy | 13 |
|  | **15** | How indeterminate index test or reference standard results were handled | 13 |
|  | **16** | How missing data on the index test and reference standard were handled | N/A |
|  | **17** | Any analyses of variability in diagnostic accuracy, distinguishing pre-specified from exploratory | 13 |
|  | **18** | Intended sample size and how it was determined | 9 |
| **RESULTS** |  |  |  |
| *Participants* | **19** | Flow of participants, using a diagram | 24 |
|  | **20** | Baseline demographic and clinical characteristics of participants | 13-14, 26-28 |
|  | **21a** | Distribution of severity of disease in those with the target condition | 13-14, 26-28 |
|  | **21b** | Distribution of alternative diagnoses in those without the target condition | 13-14 |
|  | **22** | Time interval and any clinical interventions between index test and reference standard | 10 |
| *Test results* | **23** | Cross tabulation of the index test results (or their distribution) by the results of the reference standard | 28 |
|  | **24** | Estimates of diagnostic accuracy and their precision (such as 95% confidence intervals) | 14-16, 28-29 |
|  | **25** | Any adverse events from performing the index test or the reference standard | N/A |
| **DISCUSSION** |  |  |  |
|  | **26** | Study limitations, including sources of potential bias, statistical uncertainty, and generalisability | 16-17 |
|  | **27** | Implications for practice, including the intended use and clinical role of the index test | 17-18 |
| **OTHER INFORMATION** |  |  |  |
|  | **28** | Registration number and name of registry | 13 |
|  | **29** | Where the full study protocol can be accessed | 13 |
|  | **30** | Sources of funding and other support; role of funders | 19 |

STARD 2015

### AIM

STARD stands for “Standards for Reporting Diagnostic accuracy studies”. This list of items was developed to contribute to the completeness and transparency of reporting of diagnostic accuracy studies. Authors can use the list to write informative study reports. Editors and peer-reviewers can use it to evaluate whether the information has been included in manuscripts submitted for publication.

### Explanation

A **diagnostic accuracy study** evaluates the ability of one or more medical tests to correctly classify study participants as having a **target condition.** This can be a disease, a disease stage, response or benefit from therapy, or an event or condition in the future. A medical test can be an imaging procedure, a laboratory test, elements from history and physical examination, a combination of these, or any other method for collecting information about the current health status of a patient.

The test whose accuracy is evaluated is called **index test.** A study can evaluate the accuracy of one or more index tests. Evaluating the ability of a medical test to correctly classify patients is typically done by comparing the distribution of the index test results with those of the **reference standard**. The reference standard is the best available method for establishing the presence or absence of the target condition. An accuracy study can rely on one or more reference standards.

If test results are categorized as either positive or negative, the cross tabulation of the index test results against those of the reference standard can be used to estimate the **sensitivity** of the index test (the proportion of participants *with* the target condition who have a positive index test), and its **specificity** (the proportion *without* the target condition who have a negative index test). From this cross tabulation (sometimes referred to as the contingency or “2x2” table), several other accuracy statistics can be estimated, such as the positive and negative **predictive values** of the test. Confidence intervals around estimates of accuracy can then be calculated to quantify the statistical **precision** of the measurements.

If the index test results can take more than two values, categorization of test results as positive or negative requires a **test positivity cut-off**. When multiple such cut-offs can be defined, authors can report a receiver operating characteristic (ROC) curve which graphically represents the combination of sensitivity and specificity for each possible test positivity cut-off. The **area under the ROC curve** informs in a single numerical value about the overall diagnostic accuracy of the index test.

The **intended use** of a medical test can be diagnosis, screening, staging, monitoring, surveillance, prediction or prognosis. The **clinical role** of a test explains its position relative to existing tests in the clinical pathway. A replacement test, for example, replaces an existing test. A triage test is used before an existing test; an add-on test is used after an existing test.

Besides diagnostic accuracy, several other outcomes and statistics may be relevant in the evaluation of medical tests. Medical tests can also be used to classify patients for purposes other than diagnosis, such as staging or prognosis. The STARD list was not explicitly developed for these other outcomes, statistics, and study types, although most STARD items would still apply.

### DEVELOPMENT

This STARD list was released in 2015. The 30 items were identified by an international expert group of methodologists, researchers, and editors. The guiding principle in the development of STARD was to select items that, when reported, would help readers to judge the potential for bias in the study, to appraise the applicability of the study findings and the validity of conclusions and recommendations. The list represents an update of the first version, which was published in 2003.

**Supplementary e-Table 3.** **Measures of diagnostic accuracy of lung cancer clinical histological subtypes in the derivation and validation datasets.**

|  | Control | subtypes | AUC (95% CI) | Accuracy (95% CI) | Sensitivity/Recall (95% CI) | Specificity (95% CI) | PPV/Precision (95% CI) | F-score (95% CI) |
| --- | --- | --- | --- | --- | --- | --- | --- | --- |
| Training | Health | Adenocarcinoma | 0.956 (0.948-0.963) | 0.900 (0.891-0.913) | 0.864 (0.842-0.885) | 0.923 (0.913-0.934) | 0.872 (0.853-0.891) | 0.868 (0.852-0.883) |
|  | Health | Squamous cell carcinoma | 0.939 (0.916-0.955) | 0.824 (0.804-0.842) | 0.916 (0.878-0.952) | 0.810 (0.788-0.832) | 0.426 (0.390-0.473) | 0.581 (0.544-0.627) |
|  | Health | Small-cell lung cancer | 0.953 (0.934-0.972) | 0.835 (0.821-0.854) | 0.921 (0.867-0.959) | 0.828 (0.816-0.849) | 0.306 (0.291-0.331) | 0.459 (0.445-0.483) |
| Validation | Health | Adenocarcinoma | 0.753 (0.704-0.806) | 0.676 (0.623-0.728) | 0.682 (0.620-0.744) | 0.668 (0.581-0.750) | 0.718 (0.656-0.782) | 0.698 (0.647-0.748) |
|  | Health | Squamous cell carcinoma | 0.726 (0.608-0.840) | 0.522 (0.445-0.597) | 0.737 (0.543-0.917) | 0.496 (0.419-0.589) | 0.147 (0.076-0.226) | 0.243 (0.135-0.355) |
|  | Health | Small-cell lung cancer | 0.855 (0.800-0.955) | 0.517 (0.489-0.559) | 1.000 (1.000-1.000) | 0.501 (0.472-0.536) | 0.062 (0.035-0.101) | 0.115 (0.067-0.183) |

Note: In the sensitivity analyses, we evaluated the performance of the developed model in distinguish different histological subtypes from healthy controls.

**Supplementary e-Table 4.** **Measures of diagnostic accuracy of lung cancer clinical stage in the derivation and validation datasets.**

|  | Control | Case | AUC (95% CI) | Accuracy (95% CI) | Sensitivity/Recall (95% CI) | Specificity (95% CI) | PPV/Precision (95% CI) | F-score (95% CI) |
| --- | --- | --- | --- | --- | --- | --- | --- | --- |
| Training | Health | Stage Ⅰ | 0.933 (0.917-0.951) | 0.837 (0.821-0.852) | 0.902 (0.874-0.930) | 0.820 (0.804-0.837) | 0.571 (0.533-0.611) | 0.699 (0.668-0.731) |
|  | Health | Stage Ⅰ + Ⅱ | 0.941 (0.931-0.953) | 0.862 (0.849-0.875) | 0.894 (0.866-0.922) | 0.850 (0.834-0.865) | 0.695 (0.659-0.726) | 0.782 (0.751-0.807) |
|  | Health | Stage Ⅰ + Ⅱ + Ⅲ | 0.950 (0.940-0.960) | 0.905 (0.894-0.916) | 0.836 (0.812-0.863) | 0.940 (0.930-0.952) | 0.876 (0.854-0.903) | 0.855 (0.839-0.874) |
|  | Health + Stage Ⅰ | Stage Ⅱ + Ⅲ + Ⅳ | 0.869 (0.854-0.883) | 0.794 (0.780-0.807) | 0.851 (0.821-0.875) | 0.776 (0.761-0.793) | 0.549 (0.521-0.582) | 0.667 (0.643-0.695) |
|  | Health + Stage Ⅰ +Ⅱ | Stage Ⅲ + Ⅳ | 0.836 (0.819-0.852) | 0.744 (0.728-0.757) | 0.847 (0.814-0.873) | 0.722 (0.704-0.741) | 0.389 (0.362-0.418) | 0.532 (0.503-0.563) |
|  | Health + Stage Ⅰ +Ⅱ+ Ⅲ | Stage Ⅳ | 0.806 (0.781-0.827) | 0.692 (0.675-0.708) | 0.843 (0.794-0.890) | 0.675 (0.656-0.695) | 0.221 (0.198-0.246) | 0.350 (0.320-0.381) |
| Validation | Health | Stage Ⅰ | 0.744 (0.692-0.791) | 0.645 (0.594-0.698) | 0.792 (0.729-0.857) | 0.502 (0.412-0.580) | 0.608 (0.544-0.667) | 0.687 (0.632-0.736) |
|  | Health | Stage Ⅰ + Ⅱ | 0.742 (0.683-0.799) | 0.650 (0.602-0.699) | 0.764 (0.706-0.828) | 0.533 (0.460-0.607) | 0.625 (0.551-0.689) | 0.687 (0.635-0.734) |
|  | Health | Stage Ⅰ + Ⅱ + Ⅲ | 0.751 (0.710-0.799) | 0.656 (0.605-0.704) | 0.607 (0.547-0.681) | 0.717 (0.634-0.784) | 0.730 (0.658-0.798) | 0.662 (0.610-0.721) |
|  | Health + Stage Ⅰ | Stage Ⅱ + Ⅲ + Ⅳ | 0.682 (0.612-0.760) | 0.552 (0.504-0.604) | 0.676 (0.561-0.805) | 0.529 (0.471-0.581) | 0.208 (0.154-0.263) | 0.317 (0.243-0.393) |
|  | Health + Stage Ⅰ +Ⅱ | Stage Ⅲ + Ⅳ | 0.696 (0.626-0.767) | 0.552 (0.500-0.597) | 0.698 (0.577-0.833) | 0.529 (0.472-0.583) | 0.192 (0.140-0.247) | 0.299 (0.230-0.373) |
|  | Health + Stage Ⅰ +Ⅱ+ Ⅲ | Stage Ⅳ | 0.698 (0.526-0.899) | 0.510 (0.460-0.568) | 0.715 (0.397-1.000) | 0.504 (0.444-0.558) | 0.041 (0.015-0.070) | 0.078 (0.029-0.132) |

**Supplementary e-Table 5.** **Measures of diagnostic accuracy of subgroups in the derivation and validation datasets.**

|  | groups | Subgroup | AUC (95% CI) | Accuracy (95% CI) | Sensitivity/Recall (95% CI) | Specificity (95% CI) | PPV/Precision (95% CI) | F-score (95% CI) |
| --- | --- | --- | --- | --- | --- | --- | --- | --- |
| Training | Gender | Male | 0.962 (0.952-0.970) | 0.908 (0.893-0.920) | 0.867 (0.846-0.890) | 0.951 (0.936-0.964) | 0.948 (0.934-0.963) | 0.906 (0.890-0.919) |
|  |  | Female | 0.964 (0.953-0.972) | 0.906 (0.891-0.920) | 0.876 (0.849-0.903) | 0.936 (0.907-0.961) | 0.929 (0.890-0.960) | 0.901 (0.884-0.917) |
|  | Age | <45 | 0.989 (0.981-0.996) | 0.938 (0.925-0.953) | 0.961 (0.900-1.000) | 0.936 (0.918-0.951) | 0.591 (0.500-0.675) | 0.731 (0.661-0.794) |
|  |  | ≥45 | 0.976 (0.958-0.995) | 0.916 (0.887-0.950) | 0.916 (0.857-1.000) | 0.936 (0.919-0.951) | 0.777 (0.524-0.970) | 0.822 (0.677-0.921) |
|  | Smoking | smokers | 0.965 (0.953-0.975) | 0.904 (0.878-0.924) | 0.876 (0.846-0.904) | 0.939 (0.921-0.963) | 0.927 (0.861-0.980) | 0.900 (0.867-0.929) |
|  |  | Non-smokers | 0.966 (0.958-0.977) | 0.913 (0.899-0.928) | 0.883 (0.854-0.908) | 0.931 (0.917-0.948) | 0.882 (0.857-0.910) | 0.882 (0.860-0.901) |
|  | Fasting | Fasting | 0.962 (0.952-0.971) | 0.919 (0.908-0.932) | 0.862 (0.836-0.893) | 0.945 (0.935-0.958) | 0.875 (0.850-0.902) | 0.869 (0.849-0.890) |
|  |  | No-fasting | 0.958 (0.944-0.970) | 0.902 (0.872-0.928) | 0.873 (0.843-0.898) | 0.920 (0.862-0.955) | 0.921 (0.856-0.975) | 0.896 (0.852-0.933) |
| Validation | Gender | Male | 0.729 (0.634-0.807) | 0.624 (0.533-0.689) | 0.614 (0.523-0.712) | 0.642 (0.510-0.778) | 0.729 (0.588-0.830) | 0.665 (0.561-0.734) |
|  |  | Female | 0.753 (0.654-0.824) | 0.656 (0.553-0.727) | 0.641 (0.543-0.733) | 0.679 (0.541-0.787) | 0.749 (0.627-0.833) | 0.689 (0.589-0.763) |
|  | Age | <45 | 0.779 (0.671-0.874) | 0.694 (0.609-0.783) | 0.602 (0.398-0.833) | 0.705 (0.610-0.798) | 0.206 (0.091-0.325) | 0.302 (0.158-0.453) |
|  |  | ≥45 | 0.744 (0.641-0.868) | 0.670 (0.598-0.761) | 0.624 (0.454-0.800) | 0.675 (0.532-0.800) | 0.560 (0.121-0.954) | 0.529 (0.193-0.798) |
|  | Smoking | smokers | 0.759 (0.668-0.874) | 0.664 (0.597-0.740) | 0.641 (0.537-0.754) | 0.702 (0.545-0.883) | 0.794 (0.598-0.977) | 0.705 (0.576-0.827) |
|  |  | Non-smokers | 0.734 (0.673-0.783) | 0.650 (0.588-0.711) | 0.609 (0.527-0.688) | 0.691 (0.616-0.769) | 0.667 (0.581-0.748) | 0.636 (0.562-0.695) |
|  | Fasting | Fasting | 0.575 (0.344-0.812) | 0.459 (0.346-0.617) | 0.409 (0.231-0.632) | 0.549 (0.250-0.834) | 0.625 (0.416-0.900) | 0.484 (0.319-0.706) |
|  |  | No-fasting | 0.678 (0.387-0.831) | 0.574 (0.346-0.727) | 0.543 (0.250-0.722) | 0.627 (0.362-0.800) | 0.697 (0.455-0.837) | 0.601 (0.333-0.750) |

Note: smoking groups (evaluating the model performance in ever-smoking and non-smoking groups separately).
